# Pushing the limits: a conversation with Lihong Wang

**DOI:** 10.1117/1.NPh.13.4.040401

**Published:** 2026-10-13

**Authors:** Sava Sakadžić

**Affiliations:** Massachusetts General Hospital, Harvard Medical School, Athinoula A. Martinos Center for Biomedical Imaging, Charlestown, Massachusetts, United States

## Abstract

On behalf of Neurophotonics, Sava Sakadžić interviewed Lihong Wang, Bren Professor of Medical Engineering and Electrical Engineering at Caltech.

**Figure:**
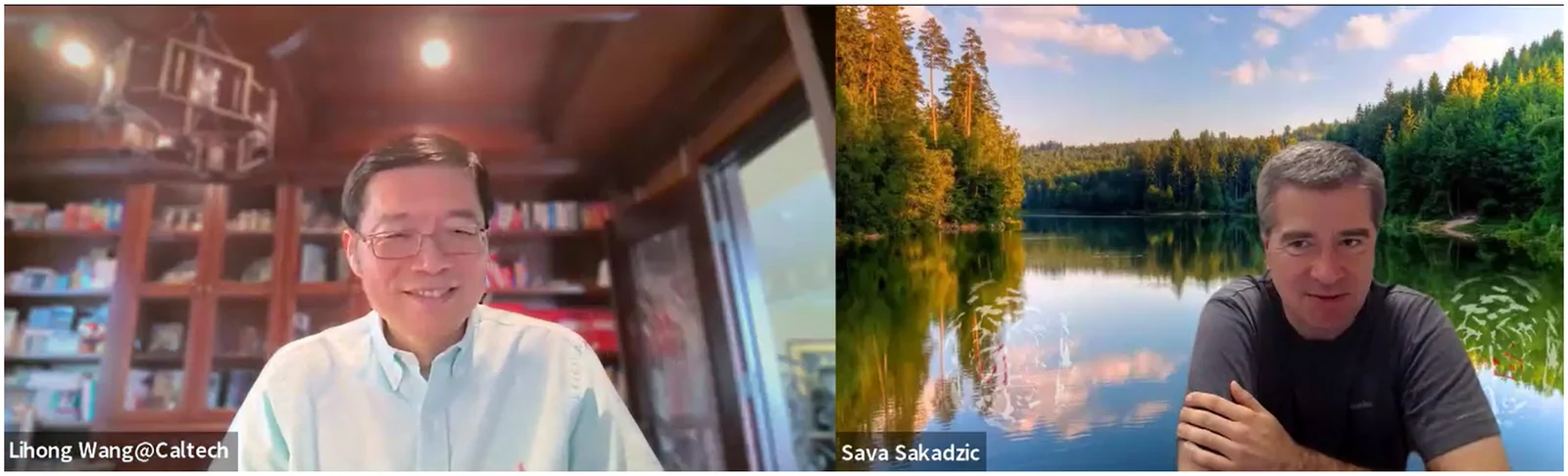
*Sava Sakadžić (right) interviewed Lihong Wang (left), Bren Professor of Medical Engineering and Electrical Engineering at Caltech, whose lab has invented or discovered functional photoacoustic tomography, 3D photoacoustic microscopy, optical-resolution photoacoustic microscopy, photoacoustic Doppler effect, photoacoustic reporter gene imaging, microwave-induced thermoacoustic tomography, universal photoacoustic reconstruction algorithm, time-reversed ultrasonically encoded optical focusing, and compressed ultrafast photography (world’s fastest camera capable of 10 trillion frames per second). View the interview video here:* https://doi.org/10.1117/1.NPh.13.4.040401

Few scientists have influenced biomedical optics as broadly as Lihong Wang. Over the course of several decades, his work has transformed our ability to see into living tissue, helped establish photoacoustic imaging as a major field, and pushed imaging technologies from the micrometer scale to phenomena occurring at the speed of light. More recently, his interests have expanded into quantum imaging and the fundamentals of quantum measurement.

When I sat down with Lihong for this *Neurophotonics* interview, I anticipated a discussion about technology. Instead, our conversation revealed a deeper theme that connects many of his contributions: a fascination with fundamental limits and an enduring curiosity about how to overcome them.

## 1 From Light Transport to Photoacoustics

Lihong’s scientific career began far from biomedical imaging. As a graduate student at Rice University, he worked with Frank Tittel, as well as Richard Smalley and Robert Curl, whose research on fullerenes would later earn a Nobel Prize. Yet rather than remain in what became the rapidly growing field of nanotechnology, he chose to move into biomedical optics at MD Anderson Cancer Center.

One of his earliest projects was developing Monte Carlo models of light transport in biological tissue. Through this work, he became deeply interested in a central challenge of optical imaging: light provides rich molecular information, but scattering severely limits how deeply we can see into tissue.

That insight led to a question that would shape much of his career: could light be combined with another form of energy that travels more effectively through tissue? Ultrasound, which scatters far less, proved to be a natural partner. The result was a series of hybrid imaging approaches and ultimately the development of photoacoustic imaging, which combines the molecular sensitivity of light with the penetration depth of sound.

## 2 Finding Opportunity at the Boundaries

As we talked, I was struck by how often Lihong returned to the idea of limits. Whether discussing optical diffusion, imaging speed, or quantum mechanics, he described research as an effort to identify important boundaries and then determine whether they can be reached, surpassed, or better understood.

This perspective helps explain why his career appears to move across such diverse fields. Work on photoacoustics led to efforts to increase imaging speed, which in turn inspired the development of ultrafast cameras capable of recording light pulses in motion. Those advances sparked an interest in quantum phenomena and eventually quantum imaging.

Although the topics changed, the motivation remained the same: pursue questions grounded in fundamental physics and explore where they lead. In his laboratory, projects are often chosen because they address major scientific constraints rather than because they fit neatly into an established discipline.

## 3 Curiosity, Learning, and Mentorship

During my years as a trainee in Lihong’s laboratory, one characteristic stood out above all others: he was always learning. His office was filled with books, new ideas regularly emerged from our discussions, and he seemed equally comfortable exploring unfamiliar territory. When I asked what motivates this constant exploration, his answer was simple: learning makes him happy.

He emphasized the importance of reading broadly, understanding foundational principles, and approaching problems from first principles. In his view, deeper understanding creates opportunities for more meaningful innovation.

That philosophy also shapes his advice to young investigators. He encourages trainees to develop ideas that are distinctly their own, by balancing established directions with bold new concepts. Equally important is learning how to choose projects wisely. Not every idea deserves pursuit, and one of the most difficult skills in science is knowing when to move on.

## 4 Looking Ahead

When asked about the future of biomedical imaging, Lihong expressed particular excitement about the convergence of imaging, robotics, and artificial intelligence. He believes that optical imaging, ultrasound, and photoacoustic methods will play increasingly important roles in guiding robotic surgeries, where real-time molecular and functional information could improve decision-making in the operating room.

After speaking with him, I was reminded that the most influential scientific careers are often driven not by a single invention, but by a way of thinking. Across fields as diverse as biomedical optics, ultrafast imaging, and quantum science, Lihong Wang has repeatedly sought out the boundaries of what is possible and asked whether they can be pushed further. That curiosity has already left a lasting mark on biomedical imaging, and it continues to point toward new frontiers.

